# Multi-omics analysis reveals Jianpi formula-derived bioactive peptide-YG-22 potentially inhibited colorectal cancer via regulating epigenetic reprogram and signal pathway regulation

**DOI:** 10.3389/fgene.2025.1560172

**Published:** 2025-03-05

**Authors:** Jun Wang, Lijuan Zhu, Yuanyuan Li, Mingming Ding, Xiyu Wang, Bo Xiong, Hongyu Chen, Lisheng Chang, Wenli Chen, Bo Han, Jun Lu, Qin Shi

**Affiliations:** ^1^ Department of Oncology, Baoshan District Hospital of Integrated Traditional Chinese and Western Medicine of Shanghai, Shanghai University of Traditional Chinese Medicine, Shanghai, China; ^2^ Department of General Surgery, Baoshan District Hospital of Integrated Traditional Chinese and Western Medicine of Shanghai, Shanghai University of Traditional Chinese Medicine, Shanghai, China; ^3^ Department of Anorectal, Shanghai Municipal Hospital of Traditional Chinese Medicine, Shanghai University of Traditional Chinese Medicine, Shanghai, China; ^4^ Department of Neurology and Institute of Neurology, Ruijin Hospital Affiliated to Shanghai Jiaotong University School of Medicine, Shanghai, China; ^5^ Department of Clinical Pharmacy, Baoshan District Hospital of Integrated Traditional Chinese and Western Medicine of Shanghai, Shanghai University of Traditional Chinese Medicine, Shanghai, China; ^6^ Key Laboratory for Translational Research and Innovative Therapeutics of Gastrointestinal Oncology, Hongqiao International Institute of Medicine, Tongren Hospital, Shanghai Jiao Tong University School of Medicine, Shanghai, China; ^7^ Shanghai Institute of Thoracic Oncology, Shanghai Chest Hospital, Shanghai Jiao Tong University School of Medicine, Shanghai, China

**Keywords:** colorectal cancer, bioactive peptide, YG-22, Jianpi formula, multi-omics

## Abstract

**Introduction:**

Colorectal cancer (CRC) is a prevalent malignancy worldwide, often treated with chemotherapy despite its limitations, including adverse effects and resistance. The traditional Chinese medicine (TCM) Jianpi formula has been demonstrated to improve efficacy of chemotherapy, however the underlying mechanisms still need to be explored. In this study, we aim to screen bioactive peptides derived from the blood of CRC patients through peptidomics and explore the molecular mechanisms of the candidate peptides in the inhibition of CRC using multi-omics analysis.

**Methods:**

In this study, we recruited 10 patients with CRC who had received either adjuvant chemotherapy or adjuvant chemotherapy combined with the traditional Chinese medicine Jianpi formula after surgery. We collected plasma samples at 2 cycles of adjuvant therapy and performed peptidomic analysis on these samples. The differentially bioactive peptides were screened using a model of HCT116 cells *in vitro*. To investigate the molecular mechanism underlying YG-22’s inhibition of the colorectal cancer cell line HCT116, we performed a multi-omics analysis, including transcriptome, metabolome, chromatin accessibility, H3K4Me3 histone methylation, and NF-κB binding site analyses.

**Results:**

Differential peptides were identified in plasma samples from patients treated with adjuvant chemotherapy combined with the Jianpi formula. Among these peptides, YG-22 exhibited the strongest cytotoxic effect on HCT116 cells, reducing cell viability in a dose- and time-dependent manner. Transcriptome analysis highlighted that YG-22 treatment in CRC modulates key pathways associated with lysosome-mediated degradation and apoptosis. Metabolomic profiling further indicated disruptions in tumor-supportive metabolic pathways. Chromatin accessibility and histone modification analyses suggested that YG-22 induces epigenetic reprogramming. Additionally, treatment with YG-22 resulted in significant changes in NF-κB binding and pathway activation.

**Conclusions:**

This study demonstrates that combining chemotherapy with TCM Jianpi formula enriches the molecular landscape and generates bioactive peptides with strong antitumor activity. Furthermore, this study also lays the foundation for further development of peptide-based therapies and highlights the value of combining traditional and modern therapeutic strategies for CRC management.

## Introduction

Colorectal cancer (CRC) ranks as the third most prevalent malignancy globally and is a leading cause of cancer-related deaths (Xi and Xu, 2021; Morgan et al., 2023; Siegel et al., 2023). Its significant incidence and mortality rates pose a major burden on healthcare systems worldwide (Xi and Xu, 2021; Alsakarneh et al., 2024; Klimeck et al., 2023). While advancements in surgery, chemotherapy, and radiotherapy have markedly improved patient outcomes, these conventional therapies are often accompanied by severe limitations (Morris et al., 2023; Feria and Times, 2024). High recurrence rates, drug resistance, and debilitating side effects—such as myelosuppression and gastrointestinal toxicity—frequently compromise their effectiveness (Adebayo et al., 2023; Al Bitar et al., 2023). Compounding these challenges is the alarming rise in CRC cases among younger populations, further underscoring the urgent need for novel therapeutic approaches that are both efficacious and less toxic (Constantinou and Constantinou, 2024).

Chemotherapy, a cornerstone of CRC treatment, continues to play a critical role in disease management (Morris et al., 2023). However, its efficacy remains limited, and its adverse effects significantly impact patients’ quality of life. In recent years, complementary and alternative medicine, particularly traditional Chinese medicine (TCM), has garnered attention as an adjunctive strategy in CRC therapy (Jiang et al., 2023; Wu et al., 2024; Lin et al., 2023; Chen et al., 2018; Chen et al., 2019; McCulloch et al., 2016). The Jianpi formula, a TCM approach rooted in holistic principles, has shown potential in addressing some of the limitations associated with chemotherapy (Zhou et al., 2019). Evidence suggests that the Jianpi formula exhibits antitumor properties, including the inhibition of tumor proliferation, induction of apoptosis, and modulation of the tumor microenvironment (He et al., 2025; Peng et al., 2018). Furthermore, it has been reported to mitigate chemotherapy-induced complications, such as neutropenia, while enhancing patients’ immune responses and overall quality of life (Zhou et al., 2019).

Despite these promising outcomes, the precise molecular mechanisms underlying the synergistic effects of chemotherapy and the Jianpi formula remain largely unexplored. This gap in knowledge has hindered the broader clinical adoption of this integrative therapeutic approach. Recent advancements in high-throughput technologies, such as peptidomics (Wang et al., 2012) and multi-omics analyses (Zhao et al., 2024), provide an opportunity to investigate these mechanisms in greater depth. Bioactive peptides, which are short amino acid sequences with regulatory functions, have been identified as critical players in various biological processes, including cancer progression and treatment response (Quintal-Bojórquez and Segura-Campos, 2021; Cui et al., 2019; Zhang et al., 2023). By screening bioactive peptides derived from the blood of CRC patients, researchers can uncover key molecular pathways influenced by these peptides. However, limited research has explored whether combining the Jianpi formula with chemotherapy alters the peptide profile in the peripheral blood of CRC patients and whether these peptides contribute to enhancing chemotherapy efficacy.

In this study, we aim to identify and characterize bioactive peptides in CRC patients’ blood using peptidomics, and to explore their biological functions in inhibiting CRC. By integrating multi-omics approaches, including transcriptomics, proteomics, and metabolomics, we seek to elucidate the molecular mechanisms through which candidate peptides exert their effects.

## Materials and methods

### Patient enrollment, grouping, treatment, and blood sample collection

This study enrolled 10 colorectal cancer (CRC) patients, divided into two treatment groups: chemotherapy alone (*n* = 5) and chemotherapy combined with Jianpi formula (*n* = 5). Chemotherapy was performed as standard postoperative adjuvant therapy. The composition of Jianpi formula including Huangqi (*Astragalus membranaceus*, 30 g), Dangshen (*Codonopsis pilosula*,12 g), Chao-baizhu (*Atractylodes macrocephala*, 12 g), Fuling (*Poria cocos,* 12 g), Zhi-gancao (*Glycyrrhiza uralensis Fisch*, 6 g), Banxia (*Pinellia ternate*, 6 g), Tianlong (*Gekko japonicus*, 6 g), Hongteng (*Caulis sargentodoxae*, 30 g), Tengligen (*Actinidia arguta*, 30 g), Sheng-muli (*Ostreae Concha*, 30 g). The formula was decocted into a solution and taken orally twice a day. The extract from the Jianpi formula has been shown to induce apoptosis in HCT116 cells, prevent HCT116 cell invasion, and inhibit HCT116 cell viability *in vitro* (data not shown). Patients’ clinical characteristics, including gender, age (54–79 years), and histopathological subtypes such as ulcerative adenocarcinoma, moderate-differentiated adenocarcinoma, poor-differentiated adenocarcinoma, and tubular adenocarcinoma, are listed in Table 1. Blood samples were collected after two cycles of therapy and were processed to isolate plasma, then stored at −80°C for peptidome analysis.

**TABLE 1:** Clinical information of the colorectal cancer patients who received chemotherapy or chemotherapy plus Jianpi formula.

Groups	Patients	Gender	Age	Types of pathology	Time of blood collection
Chemotherapy	Patient 1	Female	79	Ulcerative adenocarcinoma	2 cycles of therapy
	Patient 2	Male	74	Moderate-differentiated adenocarcinoma	2 cycles of therapy
	Patient 3	Female	54	Poor-differentiated adenocarcinoma	2 cycles of therapy
	Patient 4	Male	75	Moderate-differentiated adenocarcinoma	2 cycles of therapy
	Patient 5	Female	68	Moderate-differentiated adenocarcinoma	2 cycles of therapy
Chemotherapy + Jianpi formula	Patient 6	Female	71	Tubular adenocarcinoma	2 cycles of therapy
	Patient 7	Male	74	Ulcerative adenocarcinoma	2 cycles of therapy
	Patient 8	Female	67	Poor-differentiated adenocarcinoma	2 cycles of therapy
	Patient 9	Female	60	Poor-differentiated adenocarcinoma	2 cycles of therapy
	Patient 10	Male	71	Poor-differentiated adenocarcinoma	2 cycles of therapy

### Polypeptide extraction

Polypeptides were extracted following a rigorous multi-step protocol. The protein samples extracted from plasma were lysed using a buffer containing 8 M urea and a 1× protein inhibitor cocktail (Roche Ltd., Basel, Switzerland). Mechanical disruption was performed through three intervals of 400 s each, followed by incubation on ice for 30 min. High-speed centrifugation at 15,000 rpm for 15 min at 4°C was used to collect the supernatant. Filtration with 3 kDa ultrafiltration spin columns (Millipore, Billerica) removed high-molecular-weight proteins, retaining peptides in the 0–3 kDa range. For peptides in the 3–10 kDa range, enzymatic hydrolysis was conducted by drying the samples, redissolving them in 100 μL of 100 mM TEAB, and incubating overnight with trypsin (Promega, Madison, WI) at 37°C. Peptides were desalted using C18 Zip Tips (MonoSpin C18, GL), dried under vacuum, and stored at −80°C for mass spectrometry analysis.

### Nano-HPLC-MS/MS analysis and bioinformatic analysis

Re-dissolved peptides were analyzed using a Thermo Scientific™ Orbitrap Fusion Lumos mass spectrometer coupled to an EASY-nanoLC 1,200 system. A 3 μL sample was loaded onto a 25 cm analytical column (75 μm inner diameter, 1.9 μm resin, Dr. Maisch) and separated using a 130-min gradient. Buffer B (80% acetonitrile with 0.1% formic acid) was increased from 4% to 50% over 120 min, followed by an increase to 95% for the final 9 min. The column flow rate was maintained at 250 nL/min, and the temperature was set at 55°C. The mass spectrometer operated in data-dependent acquisition (DDA) mode, alternating between MS and MS/MS scans. Full-scan spectra were acquired at a resolution of 120,000 with an m/z range of 350–1,500, an AGC target of 8 × 10^5^, and a maximum injection time of 50 ms. Precursor ions were fragmented using higher-energy collision dissociation (HCD) with normalized collision energies of 25, 30, and 35. MS/MS spectra were acquired at a resolution of 30,000 with an AGC target of 1 × 10^5^ and a maximum injection time of 54 ms. A dynamic exclusion window of 30 s was applied to prevent repeated ion selection, ensuring comprehensive peptide profiling. Numbers of peptides unique peptides, and proteins were identified. Then, the distribution of proteins based on the number of peptides was calculated. Lastly, protein sequence coverage distribution, KEGG pathway enrichment, pearson correlation, and heatmap of differential peptides were analyzed.

### Candidate peptides screening and cell viability evaluation

After comparing the differential expressed peptides between two treatment groups: chemotherapy alone (*n* = 5) and chemotherapy combined with Jianpi formula (*n* = 5). Six candidate peptides were finally screened out, including YP-16 (YGRKKRRQRRR-GPSVP), YP-17 (YGRKKRRQRRR-OLTSGP), YG-22 (YGRKKRRQRRR-DGSPGKDGVRG), YM-22 (YGRKKRRQRRR-LGEAFDGARDM), YP-23 (YGRKKRRQRRR-MEPLGRQLTSGP), and YD-28 (YGRKKRRQRRR-EDPQGDAAOKTDTSHHD). And then, the candidate peptides were synthesized based on their amino acid sequences to evaluate their effects on HCT116 cell viability. Briefly, a total of 1,500 cells per well were seeded in 96-well plates and incubated overnight in a culture medium under appropriate conditions. Following incubation, the cells were treated with indicated peptides (5 mg/mL) at the desired concentrations for 24 h. Cell viability was then assessed using the CCK-8 assay (Dojindo, Japan), following the manufacturer’s instructions. After adding the CCK-8 reagent and incubating for the recommended time, absorbance was measured at 450 nm using a spectrophotometric plate reader (Bio-Tek, United States). Furthermore, HCT116 cells were treated with varying concentrations of YG-22 (0, 2, 4, 6, 8, and 10 mg/mL) for either 24 or 48 h to determine the IC50 values. After the incubation with YG-22 (IC50 concentration) for 48 h, the HCT116 cells were collected for subsequent analyses, including RNA sequencing (RNA-seq) to evaluate gene expression profiles, liquid chromatography-mass spectrometry (LC-MS) for metabolite analysis, assay for transposase-accessible chromatin using sequencing (ATAC-seq) to assess chromatin accessibility, and chromatin immunoprecipitation sequencing (ChIP-seq) to study the H3K4Me3 profiling and NF-κB protein-DNA interactions.

### Transcriptome analysis

For transcriptome analysis, treated and control HCT116 cells (1 × 10^5^) are collected, and total RNA is extracted using RNA isolation kit (Qiagen, Germany) following the manufacturer’s instructions. Total RNA samples were prepared with an initial concentration of at least 20 ng/μL and a total quantity of at least 2 μg, ensuring an A260/A280 ratio between 1.9 and 2.1 for quality control. mRNA was isolated using oligo-dT beads to capture polyA-tailed transcripts, followed by thermal fragmentation into 200–300 bp fragments. Reverse transcription was performed using a strand synthesis master mix to generate cDNA. Library preparation involved end-repair, A-tailing, and ligation of sequencing adapters, followed by PCR amplification and size selection for fragments of 300–400 bp, including adapter sequences. The prepared libraries underwent high-throughput sequencing on the Illumina NovaSeq 6,000 platform, producing comprehensive transcriptomic data for downstream bioinformatics analysis. Differential gene expression analysis is performed using DESeq2 or edgeR, identifying significantly up- or downregulated genes based on fold changes and adjusted *P*-values. Functional enrichment analysis, including GO and KEGG pathway analysis, is conducted to interpret biological implications, and results are visualized with heatmaps, volcano plots, and pathway diagrams.

### Metabolomics analysis

For metabolomics analysis, treated and control HCT116 cells (1 × 10^5^) are collected, washed with cold PBS, and lysed using 80% methanol to extract metabolites. The lysates are centrifuged at 12,000–15,000 × g at 4°C, and the supernatants are stored at −80°C. Metabolite profiling is performed using liquid chromatography-mass spectrometry (LC-MS) with a reverse-phase LC column and gradient elution, followed by high-resolution mass spectrometry in positive and/or negative ion modes. Data preprocessing involves peak detection, alignment, normalization, and filtering using software such as XCMS or Compound Discoverer. Metabolites are identified by matching m/z values, retention times, and fragmentation patterns to databases like HMDB or METLIN, with MS/MS used for structural confirmation. Statistical analyses, including PCA, PLS-DA, and univariate tests, are conducted to identify significantly altered metabolites, with pathway mapping performed using tools like MetaboAnalyst or KEGG Mapper. The results are visualized with heatmaps, volcano plots, and pathway diagrams, providing insights into metabolic changes induced by treatment.

### Chromatin accessibility analysis

For chromatin accessibility analysis using ATAC-seq, the process begins by lysing live HCT116 cells (5 × 10^5^) to isolate nuclei using a lysis buffer containing RSB, NP-40, Tween-20, and digitonin, followed by centrifugation to remove supernatant. The isolated nuclei are treated with a transposase mix containing Tn5 transposase at 37°C for 30 min to fragment accessible chromatin regions and insert sequencing adapters. The DNA is then purified using MinElute kits (Qiagen, Germany) and subjected to PCR amplification to optimize library quality, with purified libraries dissolved in 10 mM Tris buffer. Following library preparation, high-throughput sequencing is conducted using the Illumina NovaSeq 6,000 platform. Data processing involves quality control with FastQC, adapter trimming using Trimmomatic, genome alignment with Bowtie2, and peak calling with MACS2. Peaks are annotated using ChIPseeker and enriched motifs identified using HOMER. Differential analysis of peaks between samples is performed using DiffBind, and functional enrichment analysis is carried out to link chromatin accessibility changes to biological pathways. Results are visualized through heatmaps, volcano plots, and motif enrichment diagrams, providing insights into chromatin structure and regulatory dynamics.

### ChIP-seq analysis for H3K4Me3 profiling and NF-κB protein-DNA interactions

ChIP-seq analysis for H3K4Me3 profiling and NF-κB protein-DNA interactions was conducted using a combination of chromatin immunoprecipitation and high-throughput sequencing. HCT116 cells (5 × 10^6^) were fixed with 1% formaldehyde to crosslink proteins and DNA, followed by quenching with glycine and lysis to isolate chromatin. The chromatin was sonicated to fragment DNA, and immunoprecipitation was performed using specific antibodies for H3K4Me3 and NF-κB, coupled with Protein A + G magnetic beads. The immunoprecipitated complexes were reverse crosslinked, and DNA was purified. High-throughput sequencing libraries were prepared through end repair, A-tailing, adapter ligation, and PCR amplification, targeting fragment sizes of 100–300 bp. Sequencing was performed on the Illumina NovaSeq 6,000 platform.

Bioinformatics analysis included quality control using FastQC, adapter trimming with fastp, and alignment of clean reads to the human reference genome (hg38) using Bowtie2. Peak calling was conducted with MACS2 to identify binding sites and histone modifications, followed by annotation using ChIPseeker. Motif analysis, using MEME and HOMER, identified enriched motifs within peak regions, particularly for NF-κB binding. Functional enrichment analysis was carried out with KEGG pathways to link identified peaks to pathways. Visualization tools, such as heatmaps and Circos plots, were used to highlight binding site distribution and signal enrichment across the genome, providing insights into chromatin modifications and transcription factor interactions under experimental conditions.

### Statistical analysis

There were at least three biological replicates, excluding ATAC-seq and ChIP-seq analysis, for each group. Cell viability evaluation data were reported as means ± SEM. Student’s t-test (two-tailed) or one-way ANOVA with Bonferroni’s multiple comparison test were used. *P*-values of <0.05, or 0.01, or 0.001 were deemed significant.

## Results

### Clinical information of colorectal cancer patients enrolled in this study

The baseline clinical characteristics of colorectal cancer patients enrolled in this study are presented in Table 1. Patients were divided into two groups based on their treatment: chemotherapy alone (*n* = 5) and chemotherapy combined with Jianpi formula (*n* = 5). In the chemotherapy group, patients included a 79-year-old female with ulcerative adenocarcinoma, a 74-year-old male with moderate-differentiated adenocarcinoma, a 54-year-old female with poor-differentiated adenocarcinoma, a 75-year-old male with moderate-differentiated adenocarcinoma, and a 68-year-old female with moderate-differentiated adenocarcinoma. Similarly, in the chemotherapy plus Jianpi formula group, patients included a 71-year-old female with tubular adenocarcinoma, a 74-year-old male with ulcerative adenocarcinoma, a 67-year-old female with poor-differentiated adenocarcinoma, a 60-year-old female with poor-differentiated adenocarcinoma, and a 71-year-old male with poor-differentiated adenocarcinoma. Blood samples were collected from all patients following 2 cycles of therapy, ensuring consistent timing for peptidome analysis.

### Peptidome and lnc-peptidome analysis in colorectal cancer patients

**FIGURE 1:**
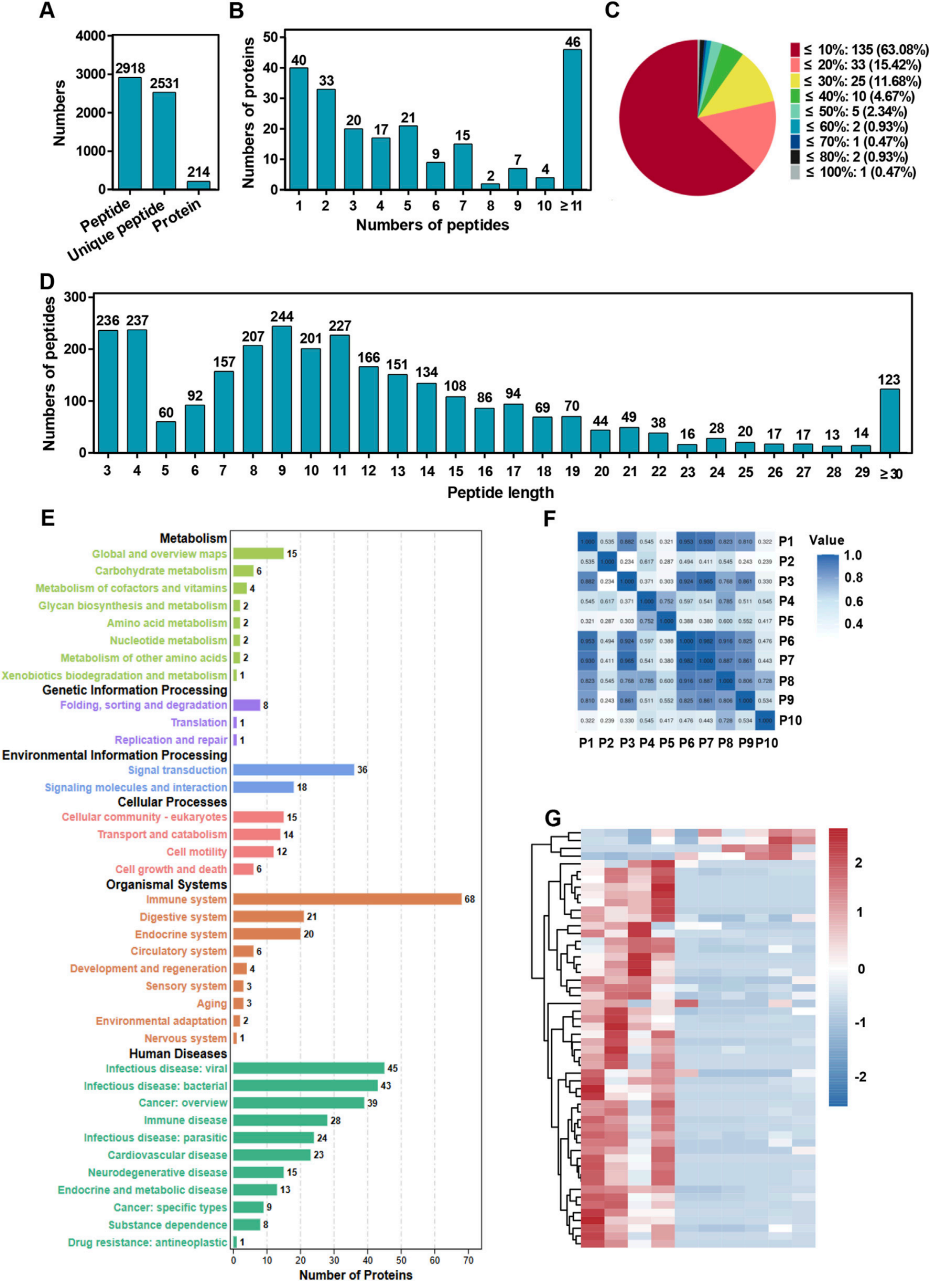
Peptidome analysis of plasma samples from colorectal cancer patients treated with chemotherapy or chemotherapy combined with the Jianpi formula. **(A)** Total number of identified peptides, unique peptides, and proteins. The bar chart summarizes the overall counts of peptides and proteins detected in the plasma samples. **(B)** Distribution of proteins based on the number of peptides identified. The histogram shows the frequency of proteins identified with varying numbers of peptides. **(C)** Protein sequence coverage distribution across the identified peptides. The pie chart illustrates the proportion of proteins categorized by sequence coverage percentages. **(D)** Protein sequence coverage distribution across the identified peptides. The pie chart illustrates the proportion of proteins categorized by sequence coverage percentages. **(E)** KEGG pathway analysis of proteins corresponding to the identified peptides. The bar chart categorizes proteins into pathways related to metabolism, genetic information processing, environmental information processing, cellular processes, organismal systems, and human diseases. The numbers on the bars represent the count of proteins associated with each category. **(F)** Pearson correlation heatmap comparing plasma sample data from colorectal cancer patients in the chemotherapy group and the chemotherapy plus Jianpi formula group. The heatmap illustrates the correlation coefficients between samples. **(G)** Heatmap of differential peptides comparing plasma sample data from colorectal cancer patients in the chemotherapy group and the chemotherapy plus Jianpi formula group. The heatmap illustrates the foldchange between samples.

The peptidome analysis conducted on plasma samples from colorectal cancer patients who had received conventional chemotherapy alone and combination chemotherapy highlighted significant differences in the protein and peptide profiles between peptidome and long non coding-peptidome (lnc-peptidome). A total of 2,918 peptides, 2,531 unique peptides and 214 proteins were identified in peptidome, while the lnc-peptidome exhibited a remarkable increase, identifying 5,115 peptides, 4,908 unique peptides and 383 proteins (Figure 1A; Supplementary Figure 1A). The distribution of these proteins based on their identified peptides showed that the majority of proteins were linked to a limited number of peptides; however, a considerable proportion difference between peptides and lnc-peptides (Figure 1B; Supplementary Figure 1B). Additionally, sequence coverage analysis revealed that while many proteins enriched in <20% coverage in peptides, while with a higher proportion showing greater than 30% coverage in lnc-peptides (Figure 1C; Supplementary Figure 1C). Notably, peptide length distribution illustrated a significant difference, with the peptides presenting a higher frequency of peptide lengths between 7 and 12 amino acids compared to a more uniform distribution in the lnc-peptides (Figure 1D; Supplementary Figure 1D).

The KEGG pathway analysis for peptides and lnc-peptides demonstrated similar enrichment patterns, with additional pathways linked to development and cellular response represented predominantly in the lnc-peptidome (Figure 1E; Supplementary Figure 1E). The heatmaps further illustrated varying expression levels of differentially expressed peptides and lnc-peptides between the treatment groups, highlighting distinct clustering patterns that suggest a functional divergence in Jianpi formula treatments (Figures 1F, G; Supplementary Figures 1F, G). These comprehensive analyses indicate that the incorporation of the Jianpi formula markedly enriches the molecular landscape in plasma samples, underscoring its potential role in enhancing therapeutic efficacy in colorectal cancer.

### Evaluation of candidate peptide effects on HCT116 cell viability and transcriptome analysis of YG-22 treatment

**FIGURE 2:**
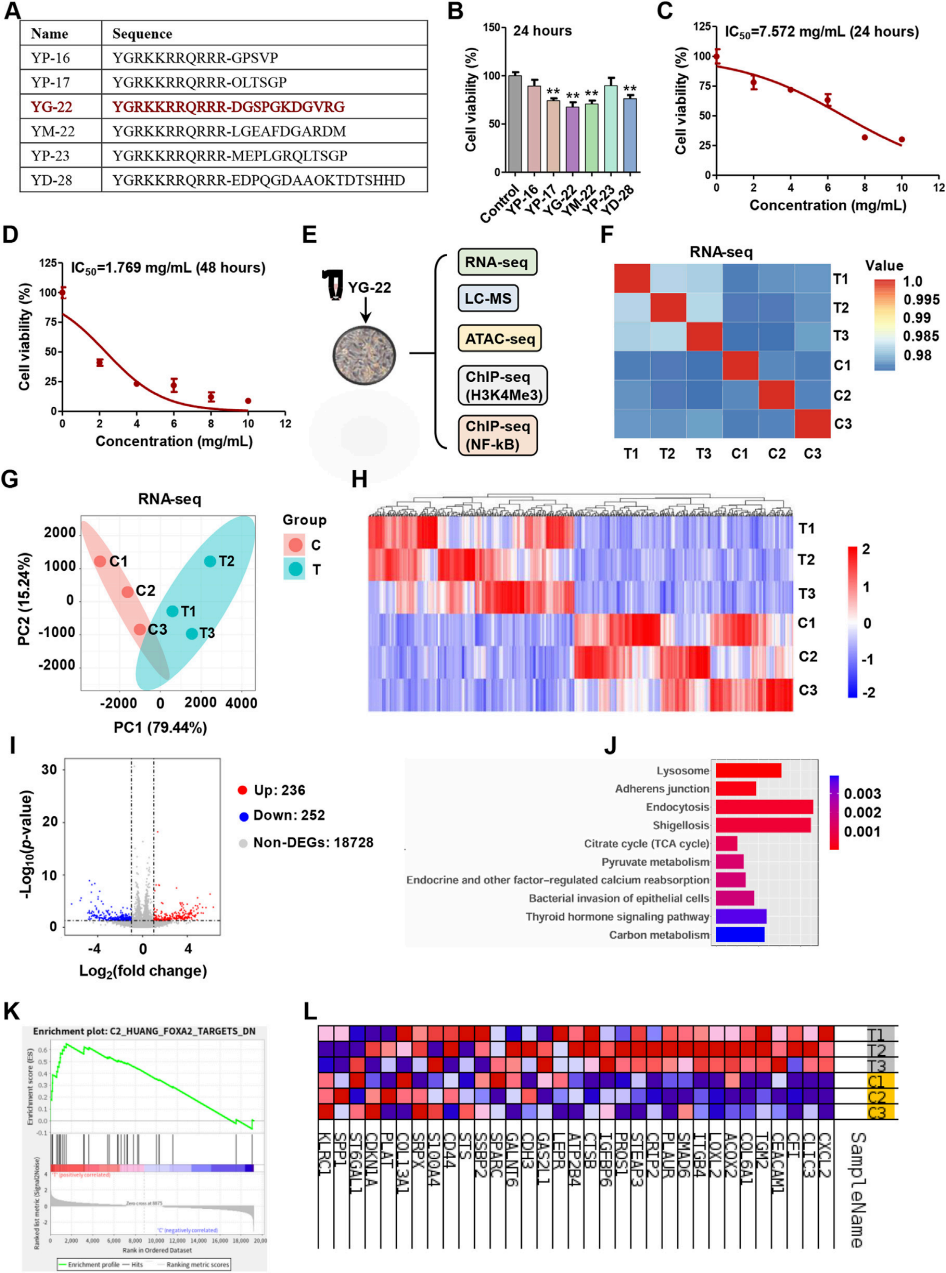
Evaluation of candidate peptide effects on HCT116 cell viability and transcriptome analysis of YG-22 treatment. **(A)** Amino acid sequences of the candidate differential peptides. The table lists the sequences for peptides YP-16, YP-17, YG-22, YM-22, YP-23, and YD-28. **(B)** Assessment of cell viability after treatment with candidate peptides (5 mg/mL) for 24 h. Data are presented as mean ± SEM (*n* = 3). **P < 0.01 indicates statistically significant differences compared to the control. **(C)** Dose-response curve showing the viability of HCT116 cells treated with YG-22 at various concentrations (2, 4, 6, 8, and 10 mg/mL) for 24 h. IC50 for 24 h = 7.572 mg/mL. Data are presented as mean ± SD (*n* = 3). **(D)** Dose-response curve showing the viability of HCT116 cells treated with YG-22 at various concentrations for 48 h. IC50 for 48 h = 1.769 mg/mL. Data are presented as mean ± SD (*n* = 3). **(E)** Schematic of multi-omics analysis performed on HCT116 cells treated with YG-22 (1.769 mg/mL) for 48 h. The analysis includes RNA-seq, LC-MS, ATAC-seq, and ChIP-seq targeting H3K4Me3 and NF-κB. **(F)** Pearson correlation heatmap comparing RNA-seq data from control (C1-C3) and YG-22-treated (T1-T3) samples. The heatmap illustrates high correlation within groups. **(G)** Principal Component Analysis (PCA) of RNA-seq data showing clear clustering between control and treated groups based on gene expression profiles. **(H)** Heatmap of differentially expressed genes (DEGs) between control and YG-22-treated groups. Red indicates upregulated genes, while blue indicates downregulated genes. **(I)** Heatmap of differentially expressed genes (DEGs) between control and YG-22-treated groups. Red indicates upregulated genes, while blue indicates downregulated genes. **(J)** KEGG pathway enrichment analysis of DEGs. Bar plots show significantly enriched pathways, with *P*-values represented by bar color intensity. **(K)** Gene Set Enrichment Analysis (GSEA) highlighting enriched pathways affected by YG-22 treatment. The plot shows pathway enrichment scores and ranks. **(L)** Heatmap of enriched differentially expressed genes involved in key pathways. Red and blue indicate higher and lower expression levels, respectively, across samples.

Among these differential peptides induced by Jianpi formula, we further screened 6 candidate peptides for cytotoxicity evaluation (Figure 2A). The analysis of candidate peptides revealed significant effects on HCT116 cell viability. The peptides, including YP-16, YP-17, YG-22, YM-22, YP-23, and YD-28, were tested at a concentration of 5 mg/mL for 24 h. Results indicated that YP-17, YG-22, YM-22 and YD-28 notably reduced cell viability, achieving statistical significance compared to the control (*P* < 0.01) (Figure 2B). Considering that YG-22 derived from collagen type I alpha 1 protein showed the best inhibitory effect on CRC cells, we then evaluated IC50 at 24 h and 48 h respectively. The dose-response curves displayed a clear relationship between YG-22 concentration and cell viability, with the 24-h IC50 determined to be 7.572 mg/mL (Figure 2C) and a reduced IC50 of 1.769 mg/mL observed at 48 h (Figure 2D). These findings suggest that YG-22 exerts a dose- and time-dependent cytotoxic effect on HCT116 cells.

To further understand the molecular mechanisms impacted by YG-22 treatment, a comprehensive multi-omics analysis was conducted, including transcriptome, metabolomics, chromatin accessibility profiling, H3K4me3 profiling and NF-κB binding profiling (Figure 2E). The transcriptome results generated a Pearson correlation heatmap, indicating a relative high degree of correlation between control and YG-22-treated groups (Figure 2F). Further Principal Component Analysis (PCA) revealed distinct clustering between the treated and control groups based on gene expression profiles (Figure 2G). Differentially expressed genes (DEGs) were identified, with illustrating 236 upregulated and 252 downregulated genes in response to YG-22 treatment (Figures 2H, I). KEGG pathway enrichment analysis highlighted significant pathways impacted by YG-22, including lysosome, adherens junction, and endocytosis pathways (Figure 2J). Gene Set Enrichment Analysis (GSEA) further supported these findings, identifying enriched pathways critical for cellular responses to YG-22 treatment (Figure 2K). A heatmap summarizing the enriched DEGs indicated distinct expression patterns across samples, providing insights into the key biological processes modulated by YG-22 (Figure 2L). These results underscore the potential therapeutic mechanisms of YG-22 in colorectal cancer treatment.

### Metabolomics analysis of HCT116 cells treated with YG-22

**FIGURE 3:**
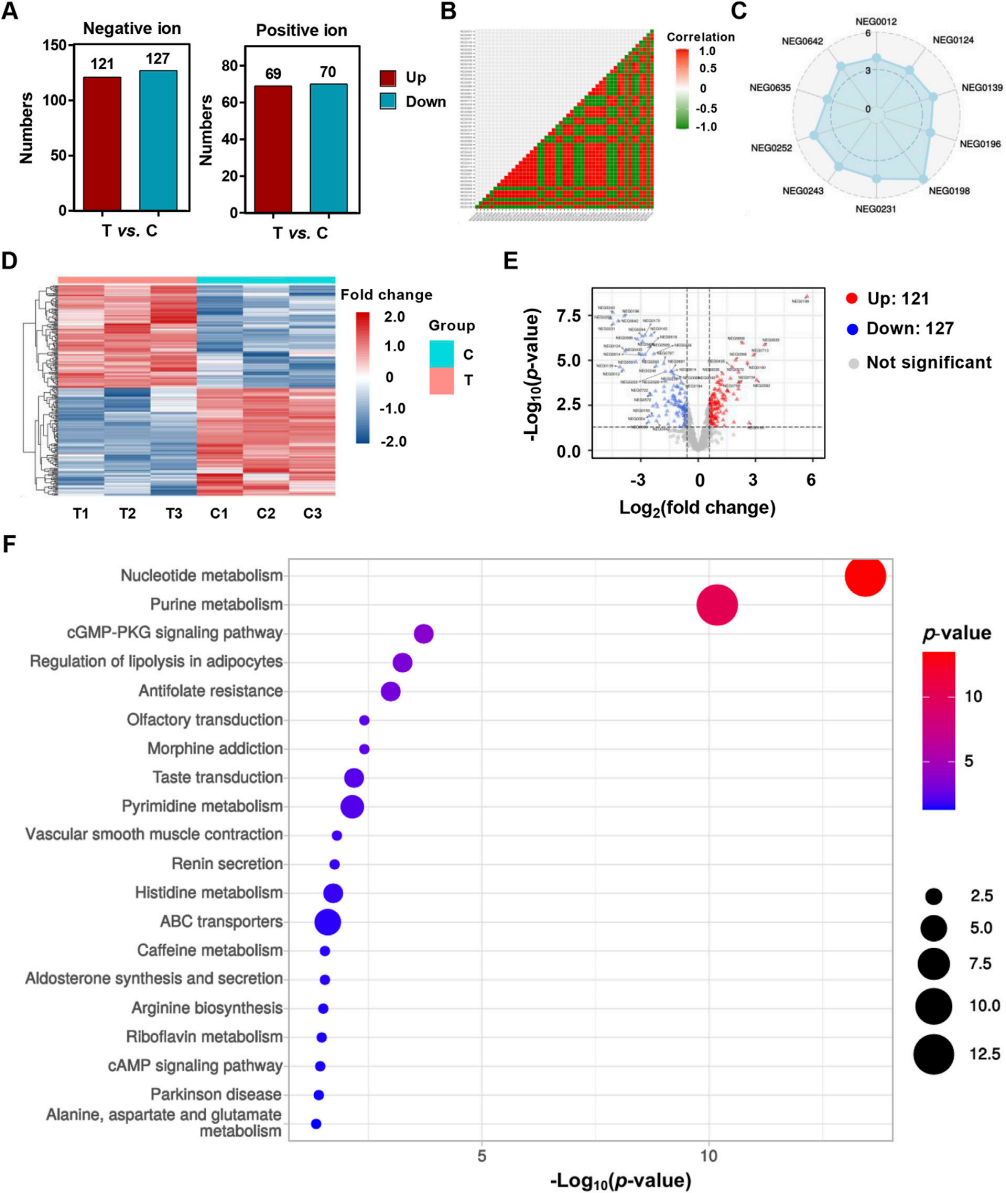
Metabolomics analysis of HCT116 cells treated with YG-22. **(A)** Total number of upregulated and downregulated metabolites identified in negative ion (NEG) and positive ion (POS) models. The bar chart shows the counts of significantly altered metabolites between treated (T) and control (C) groups. **(B)** Correlation matrix of metabolites detected in control and YG-22-treated samples based on the NEG mode. The heatmap displays correlation coefficients between samples, with red and green indicating positive and negative correlations, respectively. **(C)** Radar plot visualizing the distribution of specific metabolites detected in the NEG mode. The plot highlights the relative abundance of key metabolites across samples. **(D)** Heatmap of differentially expressed metabolites identified through NEG mode analysis. The heatmap shows hierarchical clustering and fold change in metabolite expression levels between control (C1-C3) and YG-22-treated (T1-T3) samples. **(E)** Volcano plot highlighting significant differential metabolites between control and YG-22-treated samples in NEG mode. Red dots indicate upregulated metabolites, blue dots represent downregulated metabolites, and gray dots denote non-significant changes. **(F)** KEGG pathway analysis of differentially expressed metabolites, categorizing them into metabolic pathways and biological processes. The dot plot represents pathway enrichment, with dot size indicating the number of metabolites involved and color denoting the *P*-value significance.

The metabolomics analysis of HCT116 cells treated with YG-22 highlighted significant alterations in metabolite profiles, showcasing distinct differences between the control and YG-22-treated groups. In the negative ion (NEG) mode, a total of 121 metabolites were upregulated, while 127 were downregulated, indicating notable metabolic shifts due to YG-22 treatment (Figure 3A). Correlation analyses demonstrated strong relationships among some metabolites, with a radar plot emphasizing the relative abundance of key metabolites across samples (Figure 3B). Heatmap representations illustrated hierarchical clustering of differentially expressed metabolites, reinforcing the distinctions between control and treated (Figure 3D). The volcano plot further elucidated these differences, with red dots denoting upregulated and blue dots marking downregulated metabolites, providing a clear visual of significant changes (Figure 3E). KEGG pathway analysis categorized these metabolites into various metabolic pathways, revealing important involvement in processes such as nucleotide and purine metabolism (Figure 3F).

In addition, the positive ion (POS) mode analysis complemented the findings from the NEG mode, providing further insights into the metabolic changes associated with YG-22 treatment. Similar correlation patterns were observed, reinforcing the relationships among metabolites in the control and YG-22-treated groups (Supplementary Figure 2A). The radar plots for selected metabolites in POS mode illustrated variations in abundance (Supplementary Figure 2B), while heatmap and volcano plot analyses confirmed the differential expression of metabolites, with 69 upregulated and 70 downregulated components visually represented significantly (Supplementary Figures 2C, D). The KEGG pathway analysis for this mode also highlighted a range of metabolic pathways impacted by YG-22 treatment, including ABC transporters and cAMP signaling pathways (Supplementary Figure 2E). Collectively, these analyses underscore the extensive metabolic reprogramming induced by YG-22 in HCT116 cells, highlighting critical biological processes that may contribute to its therapeutic efficacy in colorectal cancer.

### Chromatin accessibility analysis of HCT116 cells treated with YG-22

**FIGURE 4:**
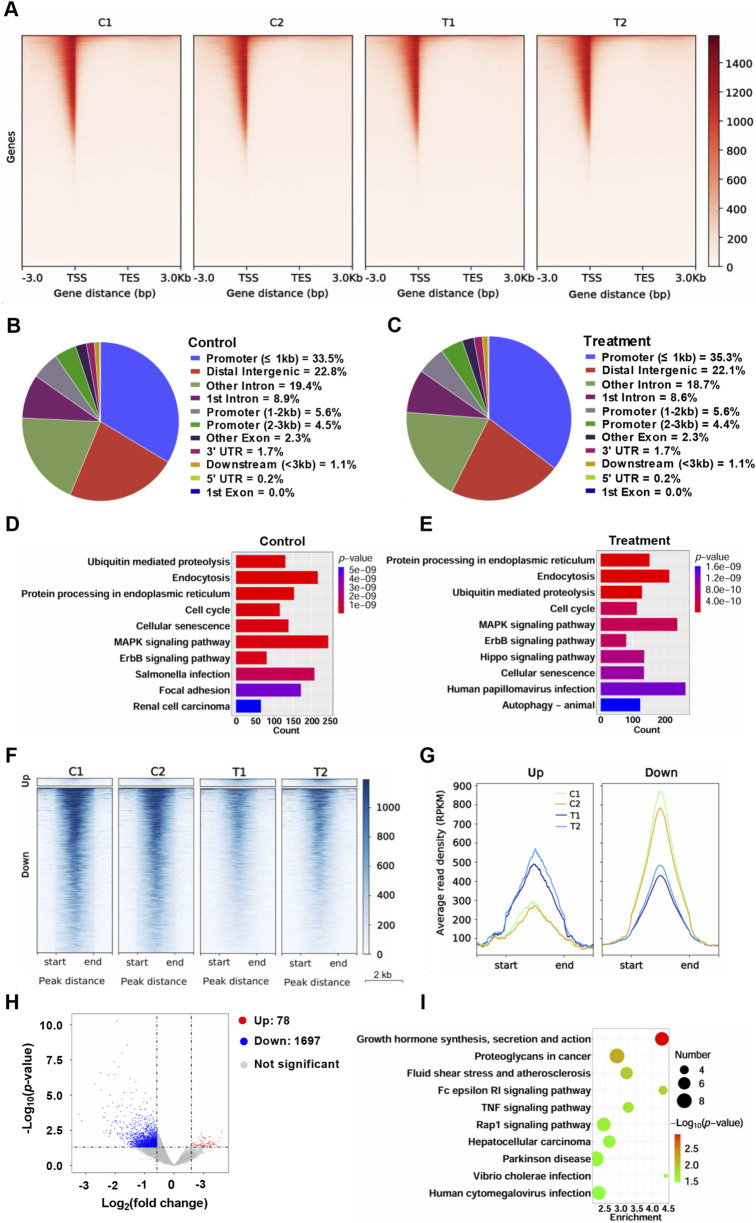
Chromatin accessibility analysis of HCT116 cells treated with YG-22. **(A)** Gene body coverage analysis across different samples. The heatmaps represent the chromatin accessibility signals distributed along gene bodies [from transcription start site (TSS) to transcription end site (TES)]. **(B)** Distribution of chromatin accessibility peaks across genomic regions in control samples. The pie chart highlights the proportion of peaks associated with various genomic features, including promoters, introns, and intergenic regions. **(C)** Distribution of chromatin accessibility peaks across genomic regions in YG-22-treated samples. The pie chart shows the peak distribution percentages across different genomic regions. **(D)** KEGG pathway analysis of promoter-associated chromatin accessibility peaks in control samples. The bar chart shows enriched pathways, with the length of the bars reflecting the number of peaks associated with each pathway and the color indicating the *P*-value significance. **(E)** KEGG pathway analysis of promoter-associated chromatin accessibility peaks in YG-22-treated samples. The enriched pathways and their significance are represented as in **(D)**. **(F)** Heatmap showing differential chromatin accessibility peaks between control and YG-22-treated samples. Clustering highlights distinct patterns of chromatin accessibility for upregulated and downregulated peaks. **(G)** Average read density (RPKM) of upregulated and downregulated peaks in control and YG-22-treated samples. Line plots show changes in chromatin accessibility signal density for each condition. **(H)** Volcano plot illustrating significant differential chromatin accessibility peaks between control and YG-22-treated samples. Red dots indicate upregulated peaks, blue dots represent downregulated peaks, and gray dots correspond to non-significant peaks. **(I)** KEGG pathway enrichment analysis for differentially accessible chromatin regions. The bubble plot represents enriched pathways, where bubble size reflects the number of peaks associated with each pathway and color indicates statistical significance (*P*-value).

To further observe the effect of YG-22 on chromatin status of HCT116 cells, we performed chromatin accessibility analysis. Chromatin accessibility analysis of HCT116 cells treated with YG-22 revealed significant differences in the chromatin landscape between control and YG-22-treated samples. Gene body coverage analysis, represented in heatmaps, indicated varied chromatin accessibility signals enriched around the transcription start site (TSS) (Figure 4A). The distribution of chromatin accessibility peaks was assessed through pie charts, highlighting the proportion of peaks associated with genomic features. In control samples, the analysis showed peaks primarily located within promoter, distal intergenic and intron (Figure 4B), while YG-22-treated samples displayed a slightly altered distribution of peaks across various regions (Figure 4C). KEGG pathway analysis demonstrated that numerous pathways were enriched in both groups, with differences in the number of accessible chromatin peaks associated with specific pathways, such as the ubiquitin-mediated proteolysis pathway and focal adhesion pathway (Figures 4D, E).

Differential chromatin accessibility between control and YG-22-treated samples was further elucidated using heatmap representations, which highlighted distinct patterns of upregulated and downregulated peaks (Figure 4F). The average read density (RPKM) analysis, indicated in line plots, illustrated changes in chromatin accessibility levels for both upregulated and downregulated peaks across conditions (Figure 4G). A volcano plot provided a visual summary of significant differential chromatin peaks, with 78 upregulated peaks and 1,697 downregulated peaks (Figure 4H). The KEGG pathway enrichment analysis for differentially accessible chromatin regions revealed that the pathways, including growth hormone synthesis, secretion and action pathway, proteoglycans in cancer pathway, were enriched significantly (Figure 4I). Supplementary analyses further clarified these findings, showcasing gene body coverage, Pearson correlation comparisons, and circular plots visualizing peak distributions across the genome (Supplementary Figure 3). This collective data underscores the extensive reconfiguration of chromatin accessibility induced by YG-22 in HCT116 cells, shedding light on potential regulatory mechanisms involved in its therapeutic effects.

### H3K4me3 profiling analysis of HCT116 cells treated with YG-22

**FIGURE 5:**
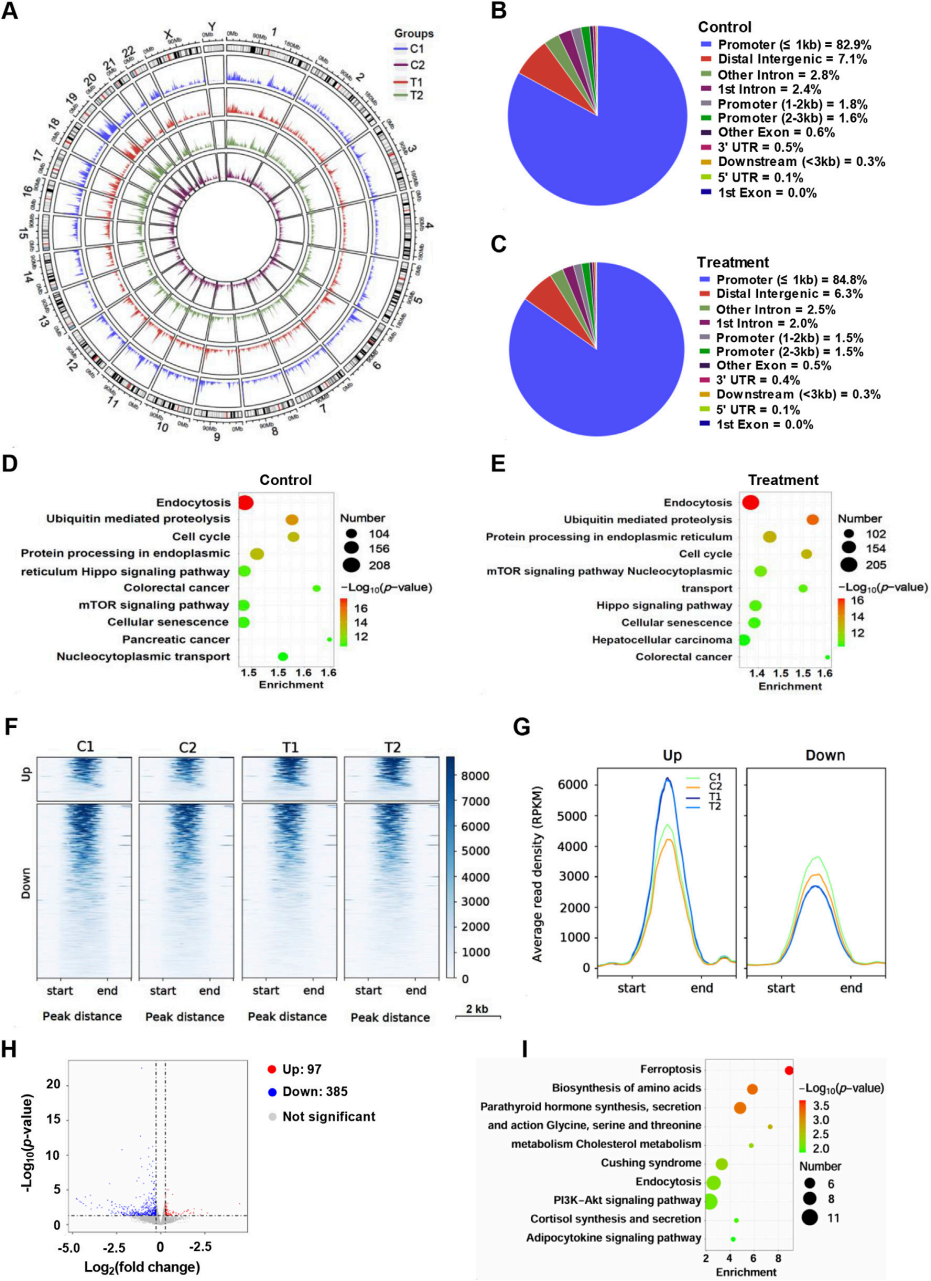
H3K4me3 profiling analysis of HCT116 cells treated with YG-22. **(A)** Circular plot visualizing the genome-wide distribution of H3K4me3 peaks across different samples (C1, C2, T1, T2). The plot highlights peak density within various genomic regions for each condition. **(B)** Distribution of H3K4me3 peaks across different chromatin regions in control samples. The pie chart represents the proportion of peaks located in promoters, introns, intergenic regions, and other genomic features. **(C)** Distribution of H3K4me3 peaks across different chromatin regions in YG-22-treated samples. The pie chart shows the genomic localization of peaks after treatment, indicating changes in distribution patterns. **(D)** KEGG pathway analysis of promoter-associated H3K4me3 peaks in control samples. The bubble plot highlights enriched pathways, with bubble size representing the number of peaks and color indicating the *P*-value. **(E)** KEGG pathway analysis of promoter-associated H3K4me3 peaks in YG-22-treated samples. Enrichment analysis reveals pathways significantly associated with treatment-related changes in H3K4me3 marks. **(F)** Heatmap showing differential H3K4me3 peaks between control and YG-22-treated samples. The clustering patterns illustrate upregulated and downregulated H3K4me3 peaks across genomic regions. **(G)** Average read density (RPKM) of upregulated and downregulated H3K4me3 peaks in control and YG-22-treated samples. Line plots display chromatin signal intensities near the differential peaks. **(H)** Average read density (RPKM) of upregulated and downregulated H3K4me3 peaks in control and YG-22-treated samples. Line plots display chromatin signal intensities near the differential peaks. **(I)** KEGG pathway enrichment analysis of differentially accessible promoter-associated H3K4me3 peaks. Bubble size reflects the number of peaks associated with each pathway, and color represents the statistical significance (*P*-value).

Furthermore, we performed ChIP-seq to analyze the H3K4Me3 profiling after YG-22 treatment upon HCT116 cells. The genome-wide distribution of H3K4me3 peaks was illustrated using a circular plot, showing peak density across various genomic regions for both control and treatment samples (Figure 5A). In control samples, 86.3% of H3K4me3 peaks were located in promoter regions, while 5.2% were found in introns and 7.1% in intergenic regions (Figure 5B). Upon treatment with YG-22, there was a slight change, with promoter-associated peaks increasing to 87.8%, intronic peaks decreasing to 4.5%, and intergenic peaks decreasing to 6.3% (Figure 5C). KEGG pathway analysis highlighted significant pathways associated with these peaks, revealing that pathways related to endocytosis and ubiquitin mediated proteolysis were enriched both in control and treatment (Figures 5D, E). Differential analysis indicated distinct clustering of upregulated and downregulated H3K4me3 modifications (Figure 5F). The average read density (RPKM) analysis, indicated in line plots, illustrated changes in H3K4me3 modification levels for both upregulated and downregulated peaks across conditions (Figure 5G). Volcano plot provided a visual summary of significant differential chromatin peaks, with 97 upregulated peaks and 385 downregulated peaks (Figure 5H). The KEGG pathway enrichment analysis for differentially H3K4me3 modification revealed that the pathways, including ferroptosis, biosynthesis of amino acids, parathyroid hormone synthesis, secretion, were enriched significantly (Figure 5I).

Supplementary analyses provided further insights into the consistency and significance of the H3K4me3 modifications. A heatmap analysis of ChIP quality confirmed the reliability of the ChIP-seq data (Supplementary Figures 4A, B). The average read density (RPKM) line plots indicated an increase in signal intensity at treatment samples (Supplementary Figure 4C). Additionally, combined circular peak distribution plots showed similar results as Figure 5A (Supplementary Figure 4D). TSS analyses further emphasized these findings, revealing that the signal density surrounding TSS regions (Supplementary Figure 4E). Collectively, these results underscore the pivotal role of H3K4me3 in modulating gene expression and illustrate the therapeutic potential of YG-22 in colorectal cancer through these alterations in chromatin modification.

### ChIP-seq analysis of NF-κB binding in HCT116 cells treated with YG-22

**FIGURE 6:**
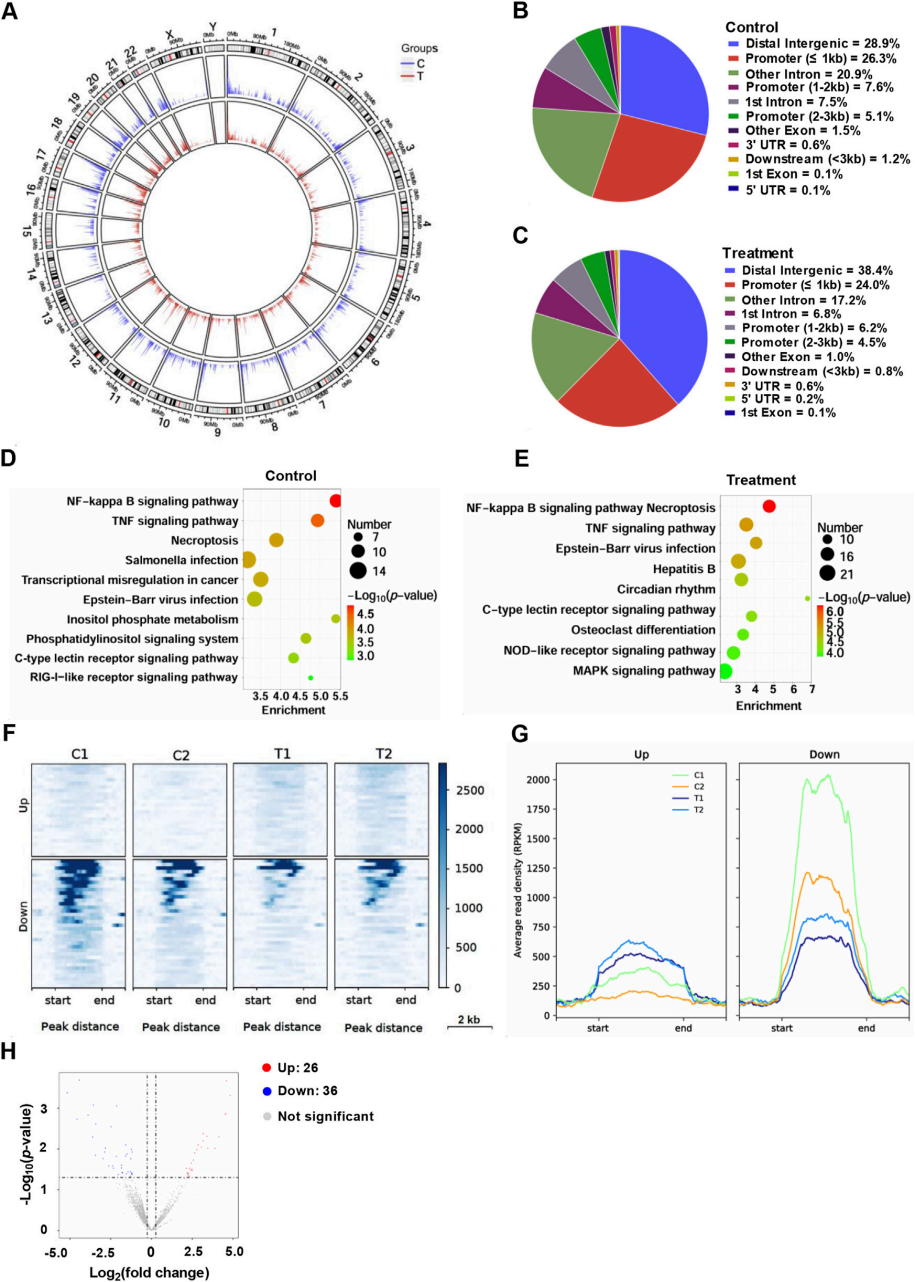
ChIP-seq analysis of NF-κB profiling in HCT116 cells treated with YG-22. **(A)** Circular plot visualizing genome-wide distribution of NF-κB peaks across different groups (control and treatment). The plot highlights the density of NF-κB binding across chromosomes. **(B)** Distribution of NF-κB peaks in various chromatin regions in control samples. The pie chart illustrates the percentage of peaks in promoters, introns, intergenic regions, and other genomic features. **(C)** Distribution of NF-κB peaks in various chromatin regions in YG-22-treated samples. The pie chart represents the proportion of peaks localized to distinct genomic regions after treatment. **(D)** KEGG pathway analysis of promoter-associated NF-κB peaks in control samples. The bubble plot shows enriched pathways, with bubble size indicating the number of peaks and color denoting statistical significance (*P*-value). **(E)** KEGG pathway analysis of promoter-associated NF-κB peaks in YG-22-treated samples. Pathway enrichment is represented similarly to panel D, highlighting pathways associated with treatment-induced changes. **(F)** Heatmap analysis showing differential NF-κB peaks between control and YG-22-treated samples. The heatmap emphasizes the clustering patterns of upregulated and downregulated peaks. **(G)** Average read density (RPKM) of upregulated and downregulated NF-κB peaks in control and YG-22-treated samples. Line plots display chromatin signal intensities near the differential peaks. **(H)** Volcano plot highlighting significant differential NF-κB peaks between control and treated samples. Red dots represent upregulated peaks, blue dots indicate downregulated peaks, and gray dots correspond to non-significant changes.

Finally, ChIP-seq analysis was conducted to investigate the genome-wide distribution. After treated with YG-22 in HCT116 cells for 48 h, enrichment of NF-κB binding peaks was collected. A circular plot revealed the NF-κB binding distribution across chromosomes, highlighting changes between control and treated groups (Figure 6A). In the control group, NF-κB peaks were predominantly located in distal intergenic regions (28.9%) and promoters (39.0%) (Figure 6B), while treatment with YG-22 led to a shift, with 38.4% of peaks observed in distal intergenic regions and a reduced percentage in promoter regions (Figure 6C). KEGG pathway analysis demonstrated significant enrichment of pathways such as “NF-kappa B signaling,” “TNF signaling,” and “Necroptosis” in the control group, with treatment further enhancing pathways like “MAPK signaling” and “C-type lectin receptor signaling” (Figures 6D, E). Heatmap analysis displayed clustering patterns of upregulated and downregulated peaks, reflecting differential NF-κB binding between the two groups (Figure 6F). The average read density (RPKM) plots illustrated the chromatin signal intensities near these peaks, highlighting significant differences between control and treated samples (Figure 6G). Finally, the volcano plot identified 26 upregulated and 36 downregulated NF-κB peaks, providing a comprehensive view of the impact of YG-22 on NF-κB activity (Figure 6H). These findings indicate that YG-22 treatment induces significant changes in NF-κB binding and pathway activation, emphasizing its potential as a modulator of NF-κB signaling.

Collectively, these data examined the peptide alterations of chemotherapy combined with the Jianpi formula in colorectal cancer patients, revealing significant changes in the peptidome and lnc-peptidome profiles, and screening the candidate bioactive peptide YG-22. Furthermore, YG-22 treatment in HCT116 cells demonstrated dose-dependent cytotoxicity, altered gene expression, metabolic reprogramming, chromatin accessibility, and significant modifications in histone and NF-κB binding, highlighting its potential as a therapeutic agent in colorectal cancer.

## Discussion

This study aimed to identify differential bioactive peptides in patients treated with chemotherapy alone and those receiving chemotherapy combined with traditional TCM-Jianpi formula. By screening these peptides, we sought to investigate their potential therapeutic effects, using *in vitro* model to evaluate cytotoxicity in HCT116 cells. The most effective peptide was then subjected to multi-omics analyses to explore its underlying mechanisms of action.

Human plasma is a vital resource in clinical and biological research, serving as a reservoir for proteins secreted by various organs. It provides insights into a patient’s physiological and pathological states and may contain biomarkers for disease detection and treatment response (Geyer et al., 2017; Xu et al., 2019). Plasma peptidomes has been used for screening novelty bioactive peptides or biomarker (Xu et al., 2019; Taguchi et al., 2021; Lu et al., 2022). In the present study, the results revealed a significant enhancement in the diversity and complexity of the plasma peptidome in patients treated with the combination therapy compared to chemotherapy alone. This suggests that the Jianpi formula not only complements chemotherapy but may also contribute to regulating key molecular pathways involved in tumor suppression, such as apoptosis and immune modulation. Among the candidate peptides identified, YG-22 exhibited the most potent cytotoxic effect, with clear dose- and time-dependent reductions in HCT116 cell viability. These findings underscore the therapeutic potential of differential peptides derived from the combined treatment approach.

Multi-omics technologies, which include transcriptomics (gene expression analysis), metabolomics (metabolic profiling), and epigenomics (chromatin and histone modification analysis), provide a comprehensive understanding of drug mechanisms (Zhao et al., 2024; Lou et al., 2022). The transcriptome primarily focuses on alterations in gene expression resulting from drug intervention, whereas metabolomics examines the impact of drug intervention on metabolites (Cui and Paules, 2010; Wilmes et al., 2013; Astarita et al., 2023; Lu et al., 2019). Chromatin accessibility analysis primarily examines the impact of drug action on chromatin accessibility and its potential influence on gene expression (Zhang et al., 2020), whereas the H3K4Me3 profiling analysis focuses on histone modifications in regions associated with active or inactive gene expression (Igolkina et al., 2019; Karlić et al., 2010). NF-κB binding can be analyzed using ChIP-seq by identifying the specific DNA regions where NF-κB transcription factors interact with the genome, providing insights into its regulatory roles in gene expression under various conditions (Mulero et al., 2019). Here, our multi-omics analysis provided valuable insights into the mechanisms of action of YG-22 in inhibiting CRC. Transcriptomic data revealed distinct gene expression changes, with enrichment in pathways related to lysosome-mediated degradation, cell adhesion, and apoptosis—processes pivotal in tumor progression and metastasis. Metabolomic profiling highlighted significant metabolic reprogramming in YG-22-treated cells, including disruptions in pathways essential for cell survival and proliferation. Furthermore, epigenomic analyses demonstrated notable alterations in chromatin accessibility and histone modifications, suggesting that YG-22 induces epigenetic reprogramming, which may enhance its antitumor effects.

These findings collectively suggest that combining TCM-Jianpi formula with chemotherapy may augment therapeutic outcomes by enriching the molecular and cellular responses to treatment. The Jianpi formula appears to amplify the generation of bioactive peptides, such as YG-22, which exhibit strong antitumor activity and target multiple pathways critical for cancer progression. This integrated approach provides a promising avenue for improving treatment efficacy. However, while this study offers valuable insights, the precise mechanisms by which the Jianpi formula enhances peptide generation and therapeutic efficacy require further investigation. Additionally, the clinical applicability and safety of the identified peptides require validation in larger clinical trials. Future studies should focus on exploring the molecular interactions of YG-22, validating its efficacy in animal models, and assessing the broader clinical implications of TCM-based combination therapies for CRC.

In conclusion, this study demonstrates the potential of TCM-Jianpi formula-derived bioactive peptides to improve colorectal cancer treatment outcomes. The identification and functional characterization of differential bioactive peptides (such as YG-22) provide a foundation for developing innovative, peptide-based therapeutic strategies.

## Data Availability

The datasets presented in this study can be found in online repositories. The names of the repository/repositories and accession number(s) can be found in NCBI database (accession numbers: OMIX008933, GSE289006, GSE289007 and GSE289008).

## Supplementary material

SUPPLEMENTARY FIGURE 1 Lnc-peptidome analysis of plasma samples from colorectal cancer patients treated with chemotherapy or chemotherapy combined with the Jianpi formula. **(A)** Total number of identified peptides, unique peptides, and proteins. The bar chart presents the overall counts of peptides and proteins detected in the plasma samples. **(B)** Distribution of proteins based on the number of peptides identified. The histogram illustrates the frequency of proteins with varying numbers of peptides detected. **(C)** Protein sequence coverage distribution across the identified peptides. The pie chart shows the proportion of proteins categorized by sequence coverage percentages. **(D)** Distribution of peptides by length. The bar chart highlights the number of peptides grouped according to their length in amino acids. **(E)** KEGG pathway analysis of proteins corresponding to the identified lnc-peptides. The bar chart categorizes these proteins into pathways related to metabolism, genetic and environmental information processing, cellular processes, organismal systems, and human diseases. The numbers indicate the count of proteins associated with each category. **(F)** Pearson correlation heatmap comparing plasma sample data from colorectal cancer patients in the chemotherapy group and the chemotherapy plus Jianpi formula group. The heatmap displays correlation coefficients between samples. **(G)** Heatmap of differentially expressed peptides between the two treatment groups. The heatmap emphasizes clustering patterns and relative expression levels of peptides, with red and blue indicating upregulated and downregulated peptides, respectively.

SUPPLEMENTARY FIGURE 2 Metabolomics analysis of HCT116 cells treated with YG-22 based on positive ion (POS) analysis. **(A)** Correlation analysis of metabolites detected in control and YG-22-treated samples based on POS mode. The heatmap shows pairwise correlation coefficients, with red indicating positive correlations and green indicating negative correlations. **(B)** Radar plot displaying the distribution of selected metabolites detected in POS mode. The plot highlights the relative abundance of key metabolites across samples. **(C)** Heatmap of differentially expressed metabolites identified through POS mode analysis. Hierarchical clustering shows differences in metabolite expression levels between control (C1- C3) and treated (T1-T3) groups. **(D)** Volcano plot illustrating significant differential metabolites between control and YG-22-treated samples in POS mode. Red dots indicate upregulated metabolites, blue dots indicate downregulated metabolites, and gray dots represent metabolites with non-significant changes. **(E)** KEGG pathway analysis of differentially expressed metabolites, categorized by metabolic pathways and biological processes. The bubble plot represents pathway enrichment, where dot size reflects the number of involved metabolites and color indicates the level of statistical significance (*P*-value).

SUPPLEMENTARY FIGURE 3 Differences in chromatin accessibility across different samples. **(A)** Average read density (RPKM) analysis for gene body coverage across different samples (C1, C2, T1, and T2). Line plots show the chromatin accessibility signal along gene bodies, from transcription start site (TSS) to transcription end site (TES). **(B)** Pearson correlation analysis comparing chromatin accessibility between control (C1 and C2) and treatment (T1 and T2) samples. Scatter plots illustrate pairwise correlations, with Pearson correlation coefficients indicated for each comparison. **(C)** TSS analysis of chromatin accessibility across different samples. Combined line plots and heatmaps depict the density of chromatin accessibility signals near the TSS regions, emphasizing differences between control and treated samples. **(D)** Circular plot visualizing chromatin accessibility peak distribution across the genome for all samples (C1, C2, T1, and T2). The plot highlights genomic regions with significant chromatin accessibility changes. **(E)** Heatmap analysis of chromatin accessibility peaks across different samples. The upper panel shows the average read density (RPKM) near peaks, while the lower panel illustrates heatmaps of peak signals, emphasizing differential chromatin accessibility clustering patterns.

SUPPLEMENTARY FIGURE 4 Differences in H3K4Me3 profiling across different samples. **(A)** Heatmap analysis of ChIP quality for both immunoprecipitated (IP) and input samples. The heatmap displays pairwise correlation coefficients among samples, emphasizing the quality and consistency of ChIP-seq data. **(B)** Pearson correlation analysis comparing H3K4Me3 profiling between control and treatment samples for both IP and input datasets. The scatter plots represent pairwise correlations, with Pearson correlation coefficients noted. **(C)** Average read density (RPKM) for IP and input samples. Line plots depict the signal intensity distribution near peak centers, comparing control and treated samples. **(D)** Circular plot visualizing the genome-wide distribution of H3K4Me3 peaks across control and treatment groups. The plot highlights differences in peak density between groups. **(E)** Transcription start site (TSS) analysis for different samples. Combined line plots and heatmaps show H3K4Me3 signal density near TSS regions, illustrating differences in chromatin accessibility between control and treated samples for both IP and input datasets.
