# Supplementary figures and images for: Multi-omics analysis reveals Jianpi formula-derived bioactive peptide-YG-22 potentially inhibited colorectal cancer via regulating epigenetic reprogram and signal pathway regulation
Source: Front Genet. 2025 Mar 5;16:1560172. doi: 10.3389/fgene.2025.1560172 (PMC11919836; doi:10.3389/fgene.2025.1560172)

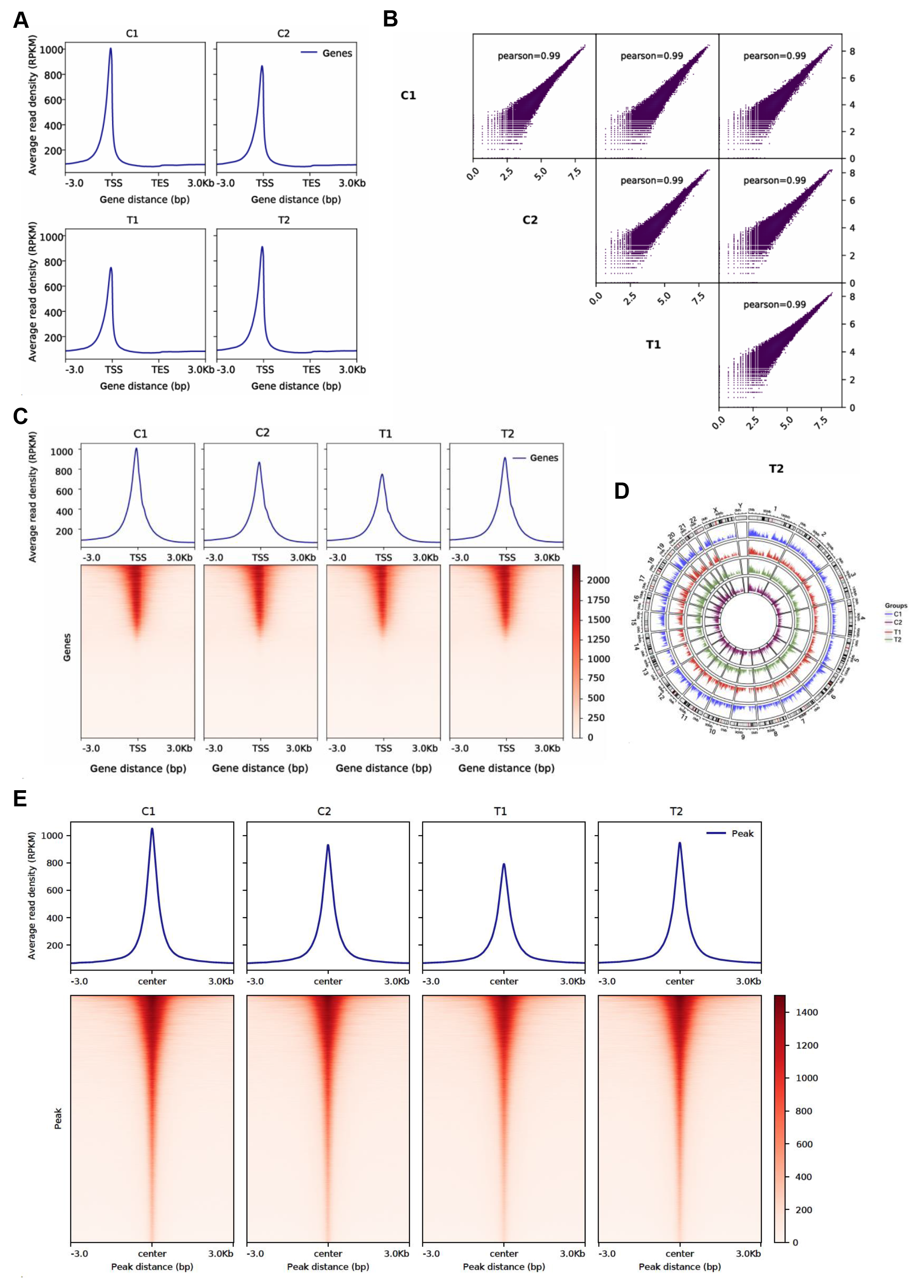

Supplement: Supplementary file 1 [file Image3.tif]

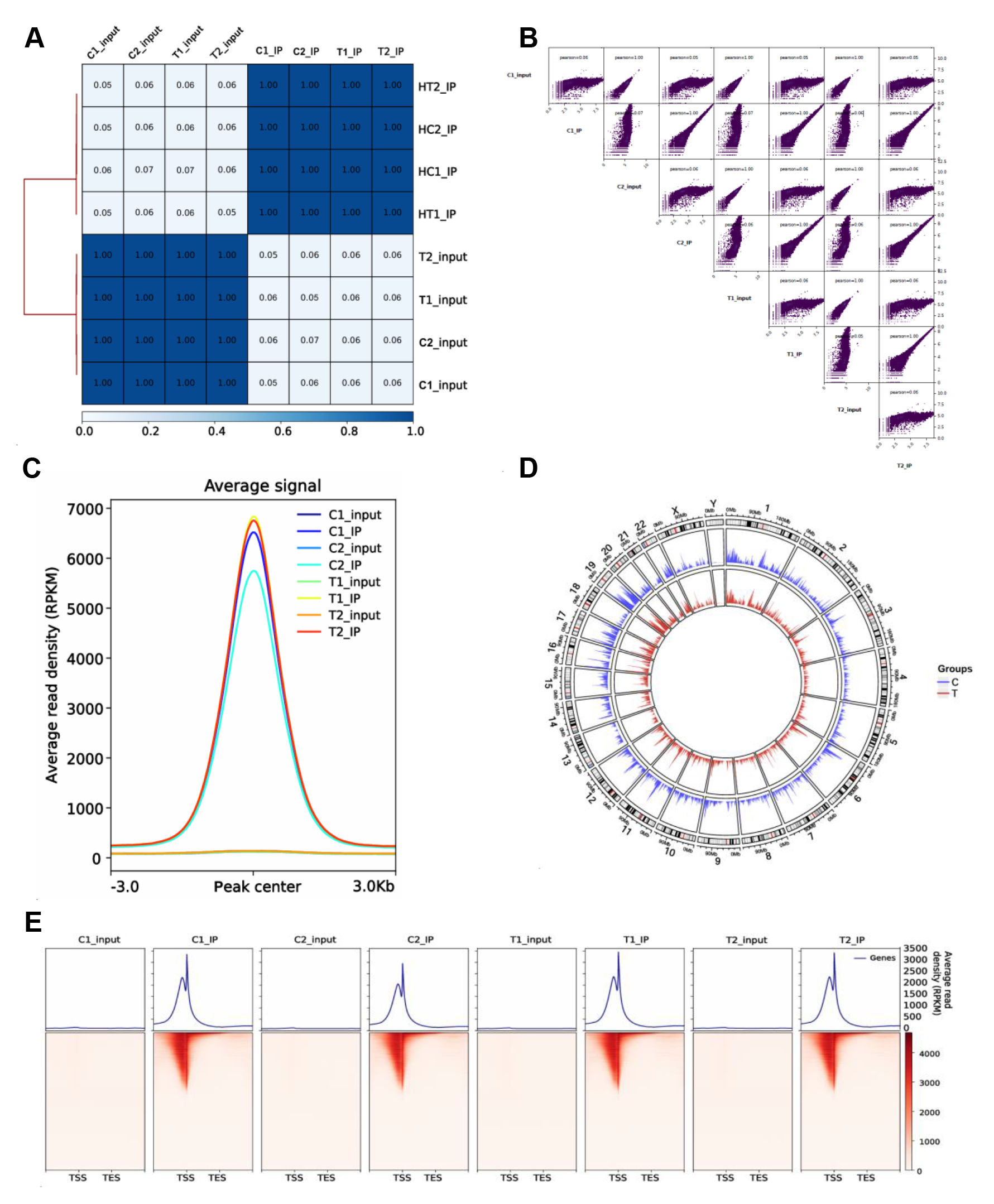

Supplement: Supplementary file 2 [file Image4.tif]

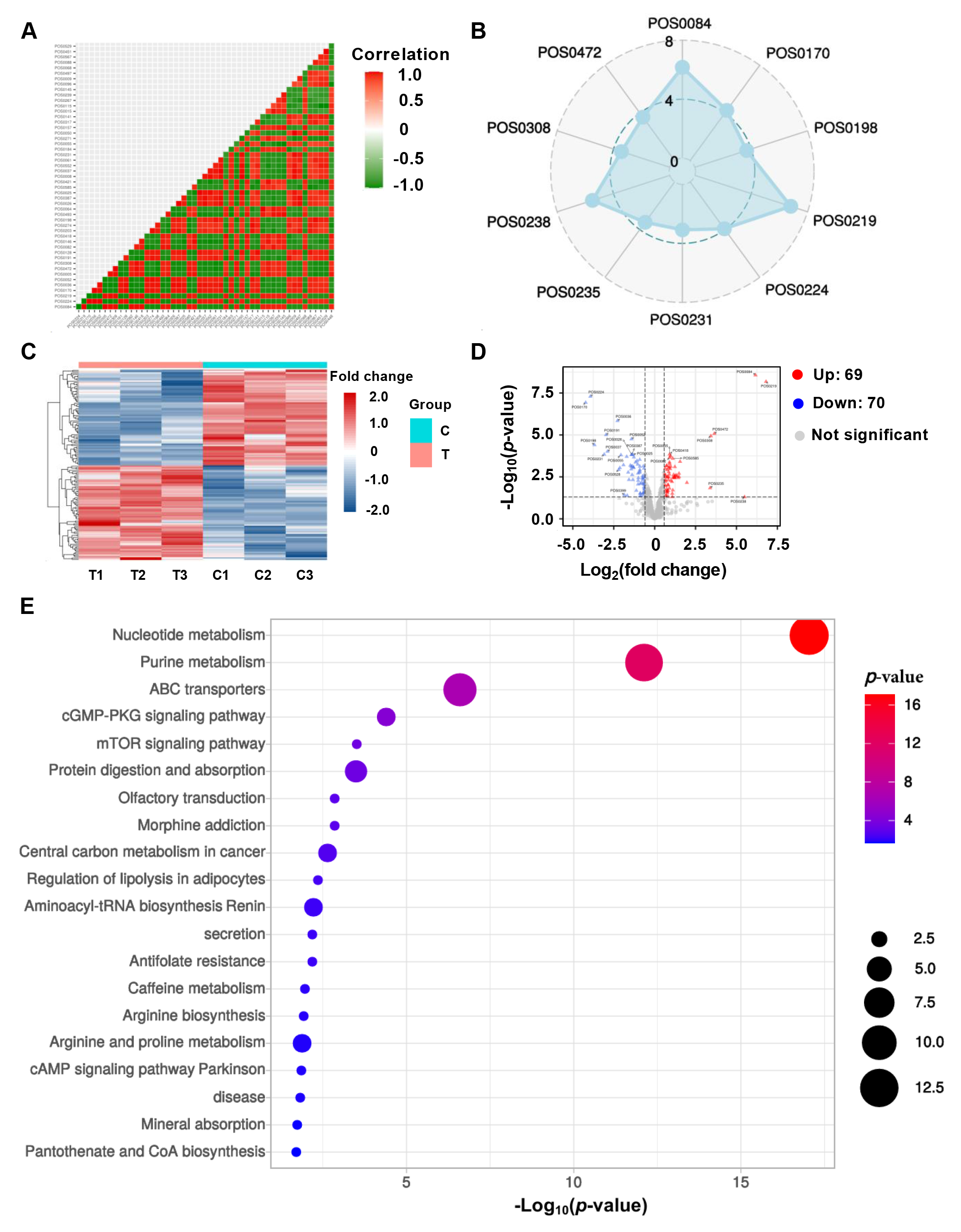

Supplement: Supplementary file 3 [file Image2.tif]

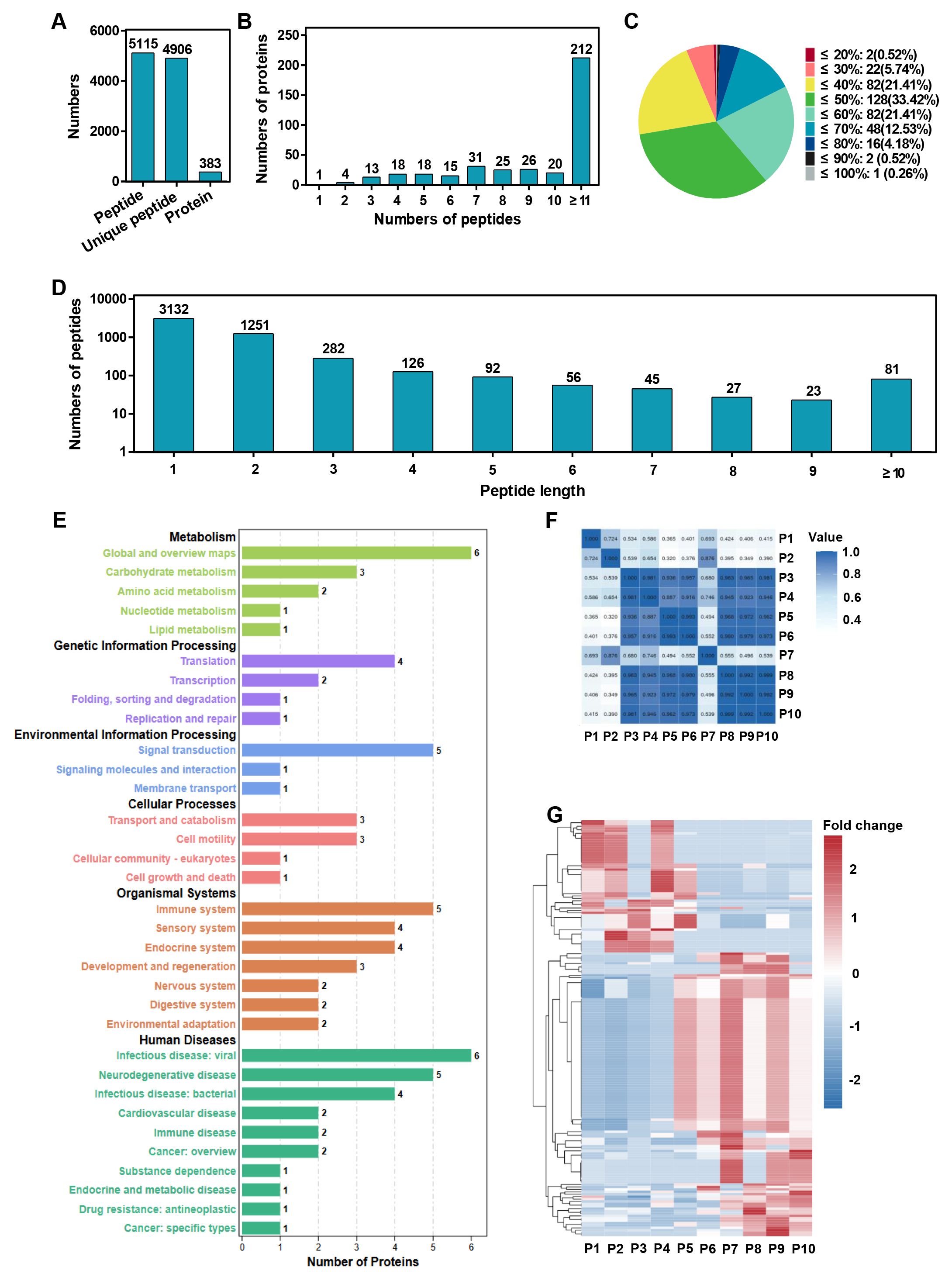

Supplement: Supplementary file 4 [file Image1.tif]
